# Cerebrovascular Events and Hospital Discharge Outcomes Associated With Drug Use: A Retrospective Case-Matched Study

**DOI:** 10.7759/cureus.50190

**Published:** 2023-12-08

**Authors:** Richard T Rogers, Ha Tran, Starlie C Belnap, Amy K Starosciak

**Affiliations:** 1 Neurology, Florida International University, Herbert Wertheim College of Medicine, Miami, USA; 2 Pediatrics, Florida International University, Herbert Wertheim College of Medicine, Miami, USA; 3 Pediatrics, Children's National Hospital, Washington, D.C., USA; 4 Miami Neuroscience Institute, Baptist Health South Florida, Miami, USA; 5 Translational Medicine, Florida International University, Herbert Wertheim College of Medicine, Miami, USA; 6 Center for Research, Baptist Health South Florida, Coral Gables, USA

**Keywords:** discharge disposition, modified rankin scale, functional outcomes in acute stroke, cerebrovascular stroke, drug use

## Abstract

Introduction

Individuals using cocaine, methamphetamines, cannabis, and other mood-altering drugs (MADs) have been reported to have greater stroke risk when compared to individuals who do not use these drugs. Yet, the impact that MAD use has on stroke outcomes has not been adequately explored, with existing research not agreeing on the extent to which the use of these drugs influences stroke outcomes. This study aimed to determine the impact that the use of common MADs has on stroke outcome factors such as length of stay (LOS), discharge modified Rankin Scale (mRS), and discharge disposition.

Methods

A retrospective case-matched study was conducted with two adult cohorts treated for cerebrovascular accidents: those who use MADs and those who do not use MADs prior to their stroke. Subjects identified for the users of MADs cohort were matched at a 1:1 ratio to those who do not use MADs (control cohort) by age, sex, and stroke type (e.g., hemorrhagic or ischemic). Logistic regression was used to calculate odds ratios for functional outcomes for stroke patients with and without prior MAD use.

Results

Active users of MADs had an increased overall risk of poor stroke outcome, i.e., increased length of stay, discharge disposition other than to home or to rehabilitation, discharge modified Rankin scale (mRS) 3-6 after controlling for stroke severity from initial National Institutes of Health Stroke Scale (NIHSS) (X^2^{9}=21.68, p<0.01, Cox adjusted R^2^=0.31). This finding was driven predominately by increased hospital LOS (p=0.006) among the MAD cohort, whereas discharge mRS 3-6 (p=0.24) and discharge disposition to care facility (p=0.27) and expired (p=0.26) did not vary significantly between groups.

Conclusion

Our study suggests that patients who had actively used MADs prior to their stroke may be at risk of poorer stroke outcomes, namely an increased LOS. Future research should be conducted to further elucidate which factors, such as individual drugs, amount of drug, acute or recreational use versus chronic or long-term use, and route of administration, for instance, specifically contribute to a longer LOS so that they may be targeted for mitigation.

## Introduction

Cerebrovascular accidents, such as hemorrhagic and ischemic stroke, contribute to the morbidity and disability associated with mood-altering drug (MAD) use [1]. The association between increased stroke risk and MADs, such as cocaine, methamphetamine, and cannabis, has been well-established in the literature [2-5]. Data regarding the use of other MADs, such as ecstasy, lysergic acid diethylamide (LSD), bath salts, synthetic marijuana (commonly known as K2 or spice), and many more, is largely limited to case reports. For instance, Boshuisen et al. described a stroke in a young patient who had recently consumed bath salts, whereas Freeman et al. observed the occurrence of stroke in two young patients who had taken “spice” [6,7]. It can be a challenge to isolate the effect a particular MAD has on stroke incidence or outcome, especially the more obscure MAD varieties, due to the prevalence of polysubstance use as well as insufficient screening methods for these particular drugs. Additionally, many of these MADs, when obtained illicitly, are often laced with other substances or impurities, which further increases the difficulty of isolating effects on stroke outcomes [8].

Importantly, much of the literature explores drug use as it relates to the risk of stroke. Fewer studies have explored drug use as it relates to stroke outcome, e.g., discharge modified Rankin Scale (mRS), length of stay (LOS), and discharge disposition. Moreover, the available studies on this topic have had mixed findings. For instance, in one study, users of crack cocaine had more favorable functional outcomes post-stroke when compared to those who did not use crack cocaine, although the authors provided no explanation for this finding [9]. A larger study based out of Iran, however, showed an increased hazard ratio of stroke mortality in users of opioids compared to those who did not use opioids, although no statistically significant functional impairments were observed between groups [10].

The current study was conducted to further expand the literature regarding drug use and stroke outcomes. Instead of examining the effect of singular drug types, and in part due to the occurrence of polysubstance abuse, this study broadly examines the impact that using MADs may have on stroke outcomes. With the ultimate goal of improving stroke management and patient counseling, the objectives of this study were to (1) characterize the stroke profiles (e.g., stroke type, comorbidities, and outcomes) of those who are using MADs at time of stroke compared to those who were not, and to (2) determine the impact that being a drug user has on the stroke outcomes of LOS, discharge mRS, and discharge disposition. We hypothesized that stroke patients with an immediate history of drug use would have worse stroke outcomes (e.g., longer LOS, higher mRS, and unfavorable discharge disposition) than those who did not use drugs. 

## Materials and methods

A retrospective case-matched study was conducted with two cohorts (those who use MADs & those who do not use MADs) treated for cerebrovascular accidents at a certified comprehensive stroke center in South Florida between October 2013 and February 2017. Subjects for both cohorts were considered eligible for the study if they (1) were between the ages of 18-89; (2) had a clinical diagnosis of stroke (ischemic or hemorrhagic), and for the MAD use cohort only; and (3) were found to be using MADs prior to presentation based on self-report or drug screen. Self-reported drugs included marijuana, cocaine, opiates, and ecstasy. Screened drugs included cannabis, cocaine, benzodiazepines, phencyclidine (PCP), and barbiturates. Subjects with only a remote history of drug use (per self-report or record review) and those not screened for drug use, unless the self-report was positive, were excluded from the analysis. Subjects included in the non-MAD use cohort had a negative drug screen and denied ever using MADs on self-report questioning. 

Subjects identified for the MAD-use cohort were matched at a 1:1 ratio to non-MAD use control cohort by age, sex, and stroke type (i.e., hemorrhagic or ischemic) in order to mitigate potential biases and confounders. Stroke data were collected for all subjects from the institution’s Get With The Guidelines (GWTG)-Stroke database. Stroke data included clinical diagnosis, stroke etiology, patient drug history, patient medical history, demographic data, length of stay (LOS), initial NIHSS, discharge disposition, and mRS at hospital discharge. Waivers of informed consent and HIPAA authorization were granted by the Baptist Health South Florida Institutional Review Board (1150294).

Descriptive statistics were used to characterize the two groups according to demographics (age and sex), comorbidities, and stroke treatment. Logistic regression with adjustment for study matching factors (e.g., age, sex, stroke type) was used to calculate odds ratios for good functional outcomes, defined as shorter LOS, mRS 0-2, and discharge disposition (i.e., home, care facility, or expired) for stroke patients with and without prior drug use. Discharge disposition was defined as a factor variable, with "expired" being the most unfavorable outcome and "to home" the most favorable. A shift analysis was used to determine the difference in the distribution of mRS scores between the two groups. All statistical tests were conducted in Statistical Package for Social Sciences (SPSS), version 27.0 (IBM Corp. Armonk, NY) at the 0.05 significance level.

## Results

MAD use findings

Using the GWTG dataset, 45 subjects were identified with a history of MAD use with 31/45 subjects undergoing drug screening at stroke presentation and 42/45 subjects providing a self-report. There were 11 subjects who had a history of MAD use documented in GWTG but did not self-report recent MAD use or test positive at the drug screen; therefore, they were excluded from further analysis. The final study sample size consisted of 34 subjects in the MAD use cohort and 34 matched controls (non-drug cohort). Of the MAD use cohort subjects, 31/34 provided positive self-report and 28/34 had positive drug screening, with all subjects in this cohort having a positive self-report and/or positive drug screen. The self-reported MAD use (n=31) results are as follows: 61% (n=19) reported marijuana use, 29% (n=9) reported cocaine use, 3% (n=1) reported opiate use, and 6% (n=2) reported ecstasy use. The drug screen (n=28) results are as follows: 79% (n=22) tested positive for cannabinoids, 36% (n=10) tested positive for cocaine, 25% (n=7) tested positive for opiates, 14% (n=4) tested positive for benzodiazepines, 4% (n=1) tested positive for PCP, and 4% (n=1) tested positive for barbiturates. Total percentages do not equal 100% due to some subjects being engaged in polysubstance use, thus testing positive for multiple MADs on drug screen. There were discrepancies between what was self-reported and what was determined by drug screen. A summary of MAD use is provided in Table 1. 

**Table 1:** MAD Use Findings Benzodiazepines, PCP, and barbiturates were not included in self-report data. Ecstasy was not included in the drug screen. Additionally, some subjects tested positive for more than one drug category, leading to totals for each drug not summing to the sample total. Discrepancies between the number of subjects and total self-report and/or screen reflect a degree of polysubstance use. Percentages were rounded to the nearest whole number. MAD = mood-altering drugs; PCP = phencyclidine

Drug Type	Self-Report % (N=31)	Drug Screen % (N=28)
Marijuana/Cannabinoids	61 (19)	79 (22)
Cocaine	29 (9)	36 (10)
Ecstasy	6 (2)	-
Opiates	3 (1)	25 (7)
Benzodiazepines	-	14 (4)
PCP	-	4 (1)
Barbiturates	-	4 (1)
Total Self-Report of Positive Screen	~100 (31)	162 (45)

Group characteristics 

Sample age and sex were case-matched, resulting in a mean age of 54 (SD=11.4) and a majority (76%) of male participants. There were fewer Hispanic subjects (35%) in the MAD use cohort compared to 59% in the control cohort, although ethnicity did not statistically vary between groups. A greater number of Black subjects were in the MAD use cohort as compared to the control cohort (χwald=4.53, p<.05), whereas a greater number of White subjects were found in the control cohort (χwald=3.96, p<.05), with other races not differing between cohorts. Other group characteristics included previous diabetes mellitus (DM) of any type, atrial fibrillation, antiplatelet or anticoagulant use, insurance status, initial NIHSS, international normalized ratio (INR), heart rate, systolic and diastolic blood pressure, and stroke treatment. Of these, only DM history (χ2=7.9, p<0.05) and insurance status (χwald=7.11, p<.05) varied significantly between cohorts. The control cohort had a higher incidence of DM history (50%, n=17) compared to the MAD use cohort (18%, n=6); whereas the MAD use cohort had a higher incidence of self-pay/Medicaid (44%, n=15) compared to controls (15%, n=5). A summary of all the group characteristics is provided in Table 2.

**Table 2:** Group Characteristics of Drug Users Versus Non-drug Users PreH = previous history; DM = diabetes mellitus type II; Afib = atrial fibrillation; INR = international normalized ratio; HR = heart rate, in beats per minute; BP = blood pressure; Tx = treatment; IV = intravenous; IA = intraarterial; M = mean; SD = standard deviation; * = statistical significance

Characteristics	Drug Group	Non-Drug Group	P-Value
Age M (SD)	54.65 (11.60)	54.88 (11.38)	Matched
Ischemic Stroke % (N)	88% (30)	88% (30)	Matched
Hemorrhagic Stroke % (N)	12% (4)	12% (4)	Matched
Male Sex % (N)	76% (26)	76% (26)	Matched
Female % (N)	24% (8)	24% (8)	Matched
Hispanic Ethnicity % (N)	38% (12)	62% (20)	0.08
Non-Hispanic Ethnicity % (N)	61% (22)	39% (14)	0.08
White/Caucasian Race % (N)	50% (17)	73% (25)	< .05*
Black Race % (N)	41% (14)	18% (6)	< .05*
Asian Race % (N)	3% (1)	3% (1)	0.75
Native American Race % (N)	3% (1)	-	0.5
Unknown Race % (N)	3% (1)	6% (2)	0.5
PreH DM % (N)	21% (6)	46% (13)	< .05*
PreH Afib % (N)	4% (1)	0% (0)	0.31
Antiplatelets or anticoagulant % (N)	18% (5)	39% (11)	0.76
Selfpay/Medicaid Insurance % (N)	43 % (12)	18% (5)	< .05*
Medicare Insurance % (N)	29% (8)	39% (11)	0.3
Private/VA Insurance % (N)	29% (8)	43% (12)	0.13
Initial NIHSS M (SD)	7.73 (8.5)	5.0 (5.9)	0.19
INR M (SD)	1.07 (0.09)	1.04 (0.08)	0.18
HR M (SD)	83.11 (18.99)	85.57 (18.99)	0.63
Systolic BP M (SD)	163 (26.35)	160.25 (27.45)	0.7
Diastolic BP M (SD)	93.82 (15.98)	92.25 (14.04)	0.7
Stroke Tx (IV-thrombolysis) % (N)	15.4% (4)	7.7% (2)	0.39
Stroke Tx (IA-thrombolysis) % (N)	8% (2)	4% (1)	0.55

Stroke outcomes between groups

**Figure 1:**
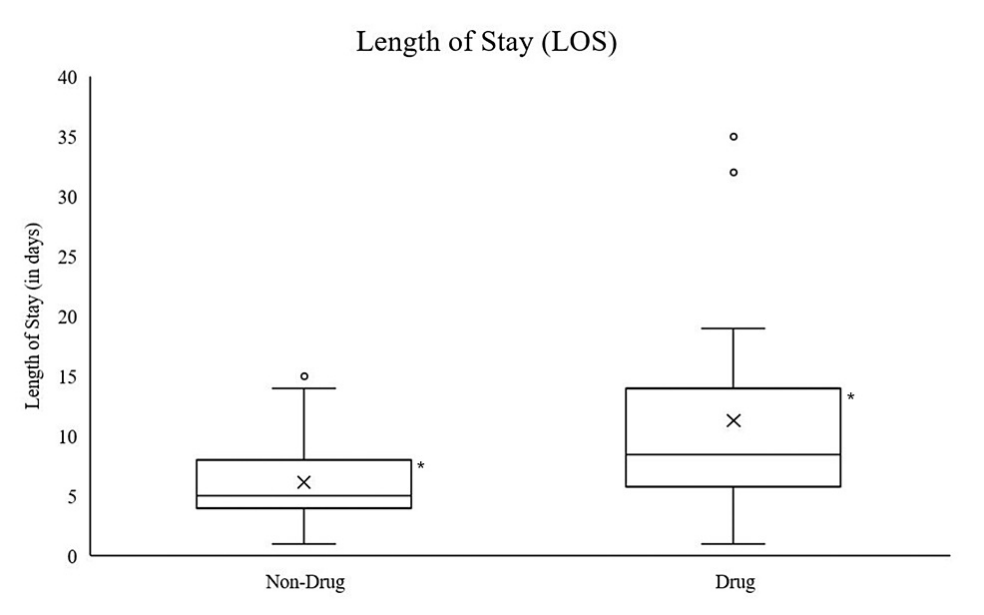
LOS Boxplot Distribution by MAD Users Versus Non-MAD Users LOS = length of stay; MAD = mood-altering drugs; X=mean; bar=median; ⁰=outliers cases; *=indicates statistical significance

The overall logistic regression model was statistically significant, with active users of MADs having an increased risk of poor stroke outcome (i.e., increased length of stay, unfavorable discharge disposition, discharge mRS 3-6) compared to the control cohort after controlling for stroke severity from initial NIHSS (X2{9}=21.68, p<0.01, Cox adjusted R2=0.31). Active users of MADs were 1.314 times more likely to have a longer length of stay compared to controls (b=0.27, Xwald{1}=6.57, 95% CI {1.07-1.60}) (Figure 1).

**Figure 2:**
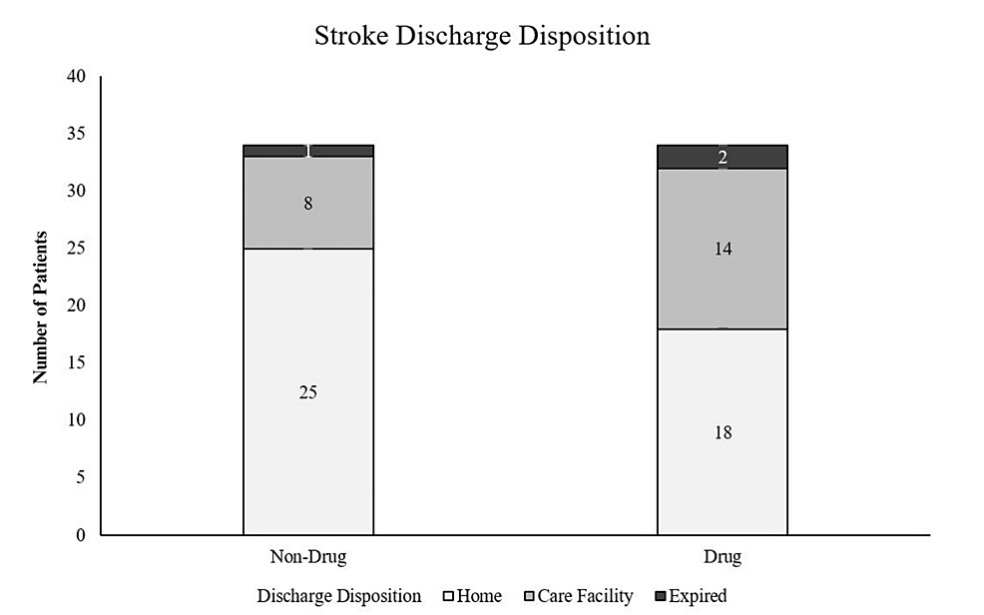
Sample Distribution for Discharge Disposition After the Stroke Event for MAD Users and Non-MAD Users MAD = mood-altering drugs not sig.

Users of MADs demonstrated a greater risk of being discharged to a care facility compared to the control cohort, although this finding was not statistically significant (Figure 2).

**Figure 3:**
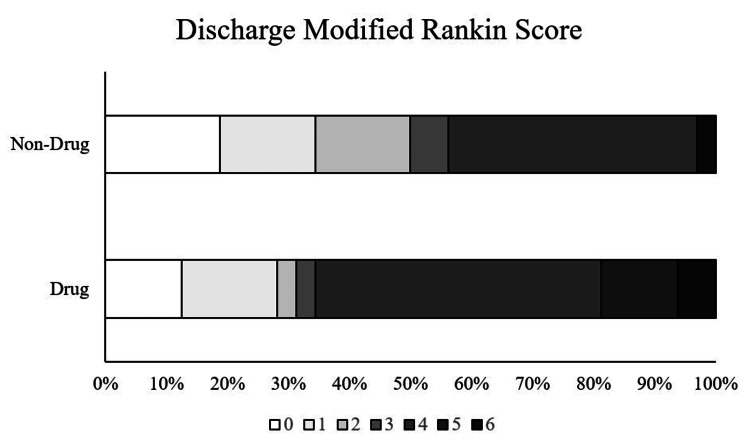
mRS at Discharge for MAD and Non-MAD Users Light gray colors = mRS values (0-2) indicating a more favorable score; dark gray color = mRS values (3-6) indicating a less favorable score. MRS = modified Rankin Scale; MAD = mood-altering drugs not sig.

Shift analysis indicated a negative shift among users of MADs, with those in the MAD use cohort being 19% more likely to have a mRS score of 3 or higher; however, this finding was not statistically significant (Figure 3).

Specifically, the MAD use cohort showed higher rates of moderate to severe disability as compared to the control cohort which may be of clinical importance. A summary of stroke outcome analysis is provided in Table 3. 

**Table 3:** Logistic Regression of Stroke Outcomes for Drug Users Versus Non-drug Users The dependent variable in this analysis is drug group coded so that 0 = non-drug group and 1 = drug group. mRS 0-2 is indicative of more favorable outcomes, while mRS 3-6 is indicative of less favorable outcomes. b = coefficient; se = standard error; MRS = modified Rankin Scale; LOS = length of stay

Independent Variable	b	se	Wald	Sig.	Odds
LOS (days)	.26	.09	7.33	.006	1.29
Home	-	-	1.67	.39	-
Care Facility	.87	.8	1.23	.27	2.38
Expired	1.75	1.55	1.28	.26	5.76
mRS (0-2)	-	-	.719	.39	
mRS (3-6)	.64	.55	1.37	.24	1.9
Constant	-2.01	.75	7.21	.007	.134
Model χ^2^ =	16.16	p. < .05	-	-	-
Pseudo R^2^ =	.32	-	-	-	-
n = 34	-	-	-	-	-

## Discussion

This study successfully characterized stroke profiles for those who used mood-altering drugs prior to their stroke (MAD use cohort) compared to those who did not (non-MAD use cohort). Those who used MADs immediately prior to stroke had a greater overall risk of poor stroke outcomes (e.g., increased LOS, higher mRS, unfavorable discharge disposition) compared to those who did not, which was mainly driven by a statistically significant increased LOS. These poor outcomes persisted after controlling for initial stroke severity, thus further isolating drug effect as the independent variable. Since increased LOS has been associated with increased mortality, increased risk of hospital-acquired infections, increased mental illness, and reduced ability to perform activities of daily living, it is important to identify stroke patients who use MADs and may be at greater risk of longer hospital stays [11].

While increased LOS has been associated with poorer patient outcomes, it is important to note that even though individuals who used MADs immediately prior to stroke experienced a longer LOS after stroke when compared to those who did not use MADs, the effects of MAD use on stroke severity may not be the sole driving factor for increased LOS. There likely are confounders unique to individuals who use MADs that impact their LOS independent of stroke severity. In fact, increased LOS has been observed in those who use MADs presenting for other medical conditions. For example, Shymon et al. demonstrated that individuals who use MADs had a longer hospital LOS compared to those who did not use MADs when presenting for orthopedic trauma [12]. This study, as well as a separate study by Levy et al., attributes this increased LOS among MAD users to difficult post-hospital placement, delays in pre-op clearance among individuals requiring surgery, and the cooccurrence of other psychosocial pathologies [12,13]. Thus in this study, it is possible that the increased LOS is due to an indirect variable related to MAD use itself (e.g., withdrawal symptoms, post-hospital placement), rather than a direct relationship between MAD use and stroke. Further research should be aimed at exploring these possible confounds.

While there are some confounders that require further exploration, one important confounder which was controlled for in this study was age. Klonoff et al. confirmed that individuals who used cocaine experienced an earlier mean age of stroke when compared to the general population [2]. Arnold et al. demonstrated that stroke patients under the age of 45 tend to have lower mRS scores and less mortality when compared to stroke patients over the age of 45 [14]. Considering such studies broadly, it can be postulated that since individuals who use MADs have an increased risk of stroke at a younger age, their functional outcomes (e.g., discharge disposition and discharge mRS) may be more favorable or at least similar compared to the average, older, non-MAD using stroke patient due to their young age alone. The current study utilized age matching to control for this possibility, and thus the use of MADs was not associated with markers of enhanced stroke recovery, as evidenced by non-significant differences between mRS and discharge disposition between groups. The removal of this potential confounder further bolsters the association between MAD use and increased LOS. 

Diabetes mellitus (DM) history and insurance status varied significantly between groups. The non-MAD use cohort had an increased proportion of subjects with DM history when compared to the MAD use cohort, while the MAD use cohort had a higher proportion of self-pay/Medicaid patients than the non-MAD use cohort. Diabetes mellitus is a well-established risk factor for stroke, with chronic hyperglycemia promoting atherosclerosis that can affect the cerebral arteries and eventually precipitate neurovascular disease [15]. Since certain drugs, such as cocaine, methamphetamine, and cannabis, have been associated with earlier onset of stroke, it may be assumed that the age-matched controls in this study would likely have higher proportions of other stroke risk factors that may have led to them having an early onset of stroke [2-5,16-18]. Thus, age-matching likely led to the discrepancy in diabetes mellitus prevalence between groups. Due to societal disparities, the higher proportion of self-pay/Medicaid patients observed in the MAD-use cohort is likely related to low socioeconomic status which is commonly associated with substance abuse [19].

A greater number of Black subjects were in the MAD use cohort compared to the non-MAD use cohort. There are many possible explanations for this observation that were not fully captured in the scope of this study, but complex extrinsic socioeconomic factors, as opposed to any intrinsic qualities, likely play a role [20]. Additionally, both the MAD use and non-MAD use cohorts had a large proportion of subjects of Hispanic ethnicity, which although representative of the South Florida community, may limit the external validity of findings to other metropolitan areas in the United States.

One other limitation of this study was the reliance on self-report and the drug screen to classify the MAD use cohort. Individuals with poor recall (e.g., dementia, altered mental status, etc.) may have erroneously denied a history of MAD use. Alternatively, some individuals may have concealed their drug use intentionally for a myriad of reasons including fear of employment consequences or social stigma. Both of these scenarios could have contributed to misclassification bias in this study. Finally, it is important to acknowledge that this study examined MAD use broadly, with no analysis of outcomes between varying drug types. Future studies should be aimed at examining the effects of individual drug classes on stroke outcomes. Furthermore, the severity of MAD use (i.e. duration, frequency, and amount) was not elucidated in this study. There may be unique differences in stroke outcomes based on the severity of MAD use as well as individuals who are chronic users versus those who use infrequently. Further analysis of these topics could be explored in further studies. 

## Conclusions

Overall, the current study suggests that patients who have actively used MADs prior to their stroke may be at risk of poorer stroke outcomes, namely an increased LOS at discharge. Future research should be conducted to further elucidate this relationship prospectively in larger, multicenter studies that may have more diverse patient populations. Comparative analyses exploring the effect that individual MADs have on stroke outcome would also help establish a foundation for understanding the pathophysiology behind drug use and stroke outcome, while further work on the relationship between use pattern (e.g., chronic versus infrequent use) would help to characterize individuals who are at highest risk. Still, the current study's broad addressment of the effect of MAD use on stroke outcomes is important, as increased LOS is associated with increased mortality and other poor outcomes for patients. The identification of MAD use in stroke patients may help clinicians anticipate longer hospital stays for these patients and allow them to make the necessary accommodations to mitigate mortality risk in these groups. 
